# Herlyn-Werner-Wunderlich Syndrome: A Rare Cause of Pelvic Pain and High CA 19-9 Levels in an Adolescent Girl

**Published:** 2016-01-01

**Authors:** Emel Unal, Hikmet Gulsah Tanyildiz, Murat Sonmezer, Hatice Gul Erkol, Suat Fitoz

**Affiliations:** 1 Department of Pediatric Hematology and Oncology, Ankara University School of Medicine, Ankara, Turkey; 2 Department of Obstetrics and Gynecology, Ankara University School of Medicine, Ankara, Turkey; 3 Department of Pediatrics, Ankara University School of Medicine, Ankara, Turkey; 4 Department of Radiology, Ankara University School of Medicine, Ankara, Turkey

**Keywords:** Mullerian duct anomaly, Renal agenesis, Herlyn-Werner-Wunderlich Syndrome, CA 19-9

## Abstract

Herlyn-Werner-Wunderlich (HWW) syndrome is a rare developmental anomaly that includes uterus didelphys with obstructed hemivagina and ipsilateral renal agenesis. A 13-year-old girl presented with chronic abdominal pain. Magnetic resonance imaging revealed uterus didelphys, hematometrocolpos and renal agenesis on the right side with imperforate hymen. Subsequently the patient was found to have Mullerian duct anomalies. CA 19-9 level was high. At laparoscopy combined with vaginoscopy hematocolpos was drained following which she improved clinically and CA 19-9 level returned to normal.

## INTRODUCTION

Development of the genitourinary organs starts at the sixth week of embryogenesis and Mullerian duct anomalies may develop during this period. Non-development or failure of fusion of the distal segments of the Mullerian ducts can lead to various types of uterine anomalies. Utero-vaginal duplication with obstructed hemivagina and ipsilateral renal agenesis is known as the Herlyn-Werner–Wunderlich syndrome.[1,2] The experience with HWW syndrome is definitely limited and consists of case reports. We report another case of HWW syndrome owing to its rarity and elevated CA 19-9 tumor marker.

## CASE REPORT

A 13-year-old female presented to her primary care physician complaining of pelvic pain and dysmenorrhea. The symptoms exaggerated over the past two months. She had experienced menarche when she was 12 year old and had a history of regular menses with cyclic pelvic pain. She had not been sexually active yet. Unilateral renal agenesis was detected incidentally during investigations for a urinary tract infection when she was two year old.

On admission to our oncology department, there was no abdominal tenderness and the external genitalia were normal. Laboratory tests revealed a normal white blood cell count and C-reactive protein. The CA19-9 level was 234.6 U/ml (normal range 0-3.5 U/ml). Alfa-fetoprotein and b-HCG levels were within the normal range. Ultrasonography revealed a right pelvic mass, agenesis of the right kidney, double uterus and blind hemivagina with hematocolpos. These findings were confirmed on MRI (Fig. 1, 2). Diagnostic laparoscopy showed retrograde menstrual flow that caused hemorrhage into the peritoneal cavity because of the blind hemivagina with hematocolpos. Following direct visualization using vaginoscopy, the bulging hemivagina was digitally located, vaginal septum was opened with a scalpel and the hematocolpos drained (Fig. 3). The blood CA 19-9 levels decreased from 234 to 84 U/ml 10 days after the laparoscopy. It further dropped to normal range during follow-up. The pain also settled postoperatively and she is doing fine on follow-up.

**Figure:**
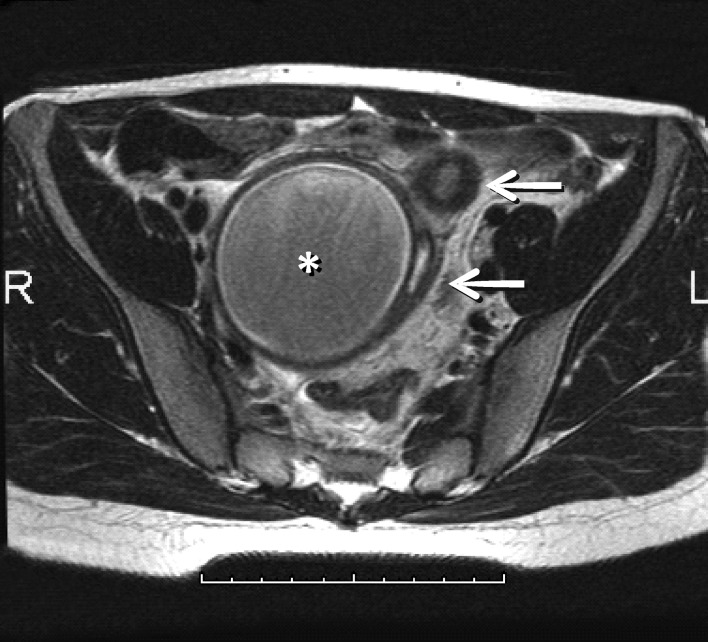
Figure 1:MRI showing dilated cavity in continuity with hydrosalpinx (arrows)

**Figure:**
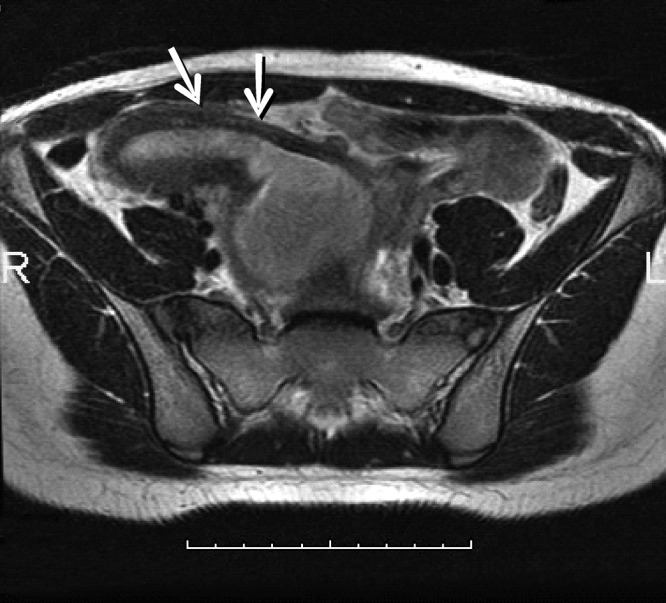
Figure 2:MRI showing dilated (right) and normal cavity (left) images of the uterus, fundus and cervix (arrows)

**Figure:**
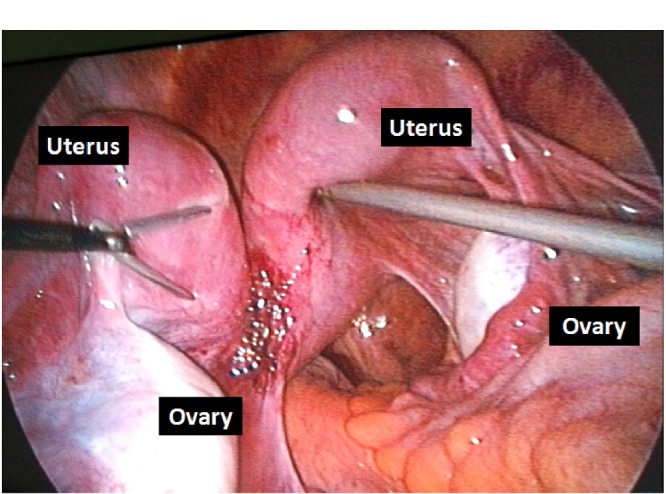
Figure 3:Laparoscopic image of the case

## DISCUSSION

Patients with the HWW syndrome are usually asymptomatic until menarche is started when they generally present with progressive and recurrent pelvic pain.[3] The retention of menstrual blood in the obstructed hemivagina after menarche can lead to the formation of a hematocolpos, which is usually clinically detected as a pelvic mass. Hemorrhage in peritoneal cavity may occur due to retrograde menstruation and endometriosis can develop, especially when the diagnosis is delayed.[4] In our patient, laparoscopy showed retrograde menstrual flow related hemorrhage in the peritoneal cavity because of the blind hemivagina with hematocolpos.

The high levels of CA 19-9 are usually encountered in pancreatic and colonic malignancies however it also raises in benign conditions such as obstructive jaundice, hepatitis, renal disorders, endometriosis, diabetic nephropathy, pleural effusion and miliary tuberculosis.[5,6] In index case high level of CA-19-9 could not be explained however normalization of CA-19-9 level after resolution of obstruction may point towards its relation with hemivaginal obstruction and hematocolpos.

Our case demonstrated the association between increased levels of CA 19-9 and congenital urogenital anomalies in adolescent girls. Another observation is that when diagnosis of renal agenesis is made in a female then genitourinary system anomalies must be ruled out as well.

## Footnotes

**Source of Support:** Nil

**Conflict of Interest:** None declared

